# Supplementary material for: Polyketide synthase gene clusters in the ericoid mycorrhizal fungus Oidiodendron maius: genome-wide prediction, expression profiling, and functional characterization of knockout mutants
Source: Front Plant Sci. 2026 Sep 16;17:1924430. doi: 10.3389/fpls.2026.1924430 (PMC13623903; doi:10.3389/fpls.2026.1924430)
Supplement: Supplementary file 1 [file SupplementaryFile1.zip › Supplementary Materials/Supplementary Folder 1.zip!/Oidma1_AssemblyScaffolds_Repeatmasked/knownclusterblast/cluster30/jgi.p_Oidma1_57780_mibig_hits.html]

| MIBiG Protein | Description | MIBiG Cluster | MiBiG Product | % ID | % Coverage | BLAST Score | E-value |
| --- | --- | --- | --- | --- | --- | --- | --- |
| AAD34555.1 | transesterase | BGC0000089 | Lovastatin\_biosynthetic\_gene\_cluster | 37 | 101.554404145 | 240 | 4.2e-63 |
| ABA02244.1 | transesterase | BGC0000098 | Monacolin\_K\_biosynthetic\_gene\_cluster | 37 | 92.7461139896 | 223 | 4.1e-58 |
